# A Novel Device-Integrated Drug Delivery System for Local Inhibition of Urinary Tract Infection

**DOI:** 10.3389/fmicb.2021.685698

**Published:** 2021-06-25

**Authors:** Kristian Stærk, Rasmus Birkholm Grønnemose, Yaseelan Palarasah, Hans Jørn Kolmos, Lars Lund, Martin Alm, Peter Thomsen, Thomas Emil Andersen

**Affiliations:** ^1^Research Unit of Clinical Microbiology, University of Southern Denmark and Odense University Hospital, Odense, Denmark; ^2^Department of Cancer and Inflammation Research, University of Southern Denmark, Odense, Denmark; ^3^Research Unit of Urology, Department of Clinical Research, University of Southern Denmark, Odense, Denmark; ^4^Biomodics ApS, Rødovre, Denmark

**Keywords:** uropathogenic *Escherichia coli*, CAUTI, large animal model, interpenetrating polymer network, urinary catheter, drug release

## Abstract

**Background:** Catheter-associated urinary tract infection (CAUTI) is a frequent community-acquired infection and the most common nosocomial infection. Here, we developed a novel antimicrobial catheter concept that utilizes a silicone-based interpenetrating polymer network (IPN) as balloon material to facilitate a topical slow-release prophylaxis of antibacterial agents across the balloon to the urinary bladder.

**Methods:** The balloon material was achieved by modifying low shore hardness silicone tubes with a hydrogel interpenetrating polymer in supercritical CO_2_ using the sequential method. Release properties and antibacterial efficacy of the IPN balloon treatment concept was investigated *in vitro* and in a porcine CAUTI model developed for the study. In the latter, Bactiguard Infection Protection (BIP) Foley catheters were also assessed to enable benchmark with the traditional antimicrobial coating principle.

**Results:** Uropathogenic *Escherichia coli* was undetectable in urinary bladders and on retrieved catheters in the IPN treatment group as compared to control that revealed significant bacteriuria (>10^5^ colony forming units/ml) as well as catheter-associated biofilm. The BIP catheters failed to prevent *E. coli* colonization of the bladder but significantly reduced catheter biofilm formation compared to the control.

**Conclusion:** The IPN-catheter concept provides a novel, promising delivery route for local treatment in the urinary tract.

## Introduction

Since the introduction of Foley catheters in the 1930s, they have become the most frequently deployed medical device with an estimated >30,000,000 implants per year in the US alone (Stickler, 2014). Unfortunately, the presence of a urinary catheter prompts a significant vulnerability to resilient catheter-associated urinary tract infections (CAUTIs) with the risk of hospitalization due to ascending or bloodstream dissemination (Stickler, 2014). The infection rate is estimated to increase by 3–7% per day in catheterized individuals (Babich et al., 2018; Ramstedt et al., 2019). With aging being one of the leading causes of urological disorders such as urinary incontinence, the constant increase in the geriatric population makes CAUTIs a growing concern (Yoo and Spencer, 2018).

The development of a CAUTI begins as the surface of the indwelling urinary catheter becomes colonized with a dense bacterial biofilm after the catheter has been in place *in situ* for some time (Stickler, 2014). In the biofilm, the bacteria are embedded in an extracellular matrix, which protects them against host defense mechanisms as well as antibiotics (Ramstedt et al., 2019).

The most common etiological agents of CAUTIs are bacteria of the Enterobacteriaceae family, particularly uropathogenic *Escherichia coli* (UPEC) (Ronald, 2003). When growing as a biofilm population, UPEC upregulates important virulence factors that are pivotal for successful infection of the bladder, and eventually, the biofilm disseminates to the bladder urine and mucosa by seeding virulent planktonic bacteria, thereby causing a symptomatic infection (Connell et al., 1996; Reisner et al., 2014; Stærk et al., 2016).

Treatment of CAUTI is difficult, since bacterial biofilms are resistant to antimicrobial therapy due to decreased penetration of antimicrobials through the biofilm matrix and the general differentiation of bacteria into less metabolically active phenotypes with decreased susceptibility to bacteriostatic agents (Singh et al., 2017). Therefore, key management strategies involve careful hygiene procedures to prevent initial contamination of the catheter surface that may seed infectious biofilms (Trautner, 2010). To further prevent bacterial biofilm formation, numerous attempts have been made to develop catheters that are coated with antibacterial substances such as silver ions, antibiotics, and noble metal alloy (NMA) in order to kill attaching bacteria or attenuate their attachment (Singha et al., 2017; Zizovic, 2020). Many of these interventions appear promising in laboratory testing but shows little or no effect in clinical trials (Pickard et al., 2012b; Aljohi et al., 2016). This failure could be in part explained by the difficulty of incorporating a stable coating with an adequate drug reservoir without compromising the favorable mechanical and biocompatible properties of the catheter polymer material.

Interpenetrating polymer networks (IPNs) are composite materials with advantageous properties from multiple polymers (Aminabhavi et al., 2015). They have been explored over the past 80 years as components in drug carrier systems and found use in microscale capsule-based formulations and biodegradable implants that enable site-specific controlled drug delivery (Changez et al., 2004, 2005; Lohani et al., 2014; Aminabhavi et al., 2015). Recently, we showed that IPNs could also successfully be incorporated into silicone-based medical devices, essentially converting the device bulk material into a drug reservoir while maintaining device functionality (Stenger et al., 2016; Klein et al., 2017). This provides a conceptual alternative solution to the current challenges of designing anti-infective coatings (Myung et al., 2008; Stenger et al., 2016).

While drug coatings or drug-loaded IPN materials are suitable for highly localized effects such as prevention of device biofilms, in the case of antimicrobial urinary devices, such functionality is often inadequate. Here, coatings are challenged by the harsh chemical urine milieu and hydrodynamic conditions, and the ubiquitous presence of microorganisms leads to recolonization of the urinary catheter and CAUTI once the drug reservoir is depleted. To effectively prevent CAUTI, a constant, preferably zero-order release of adequate drug is desirable from a large local drug reservoir.

**FIGURE 1:**
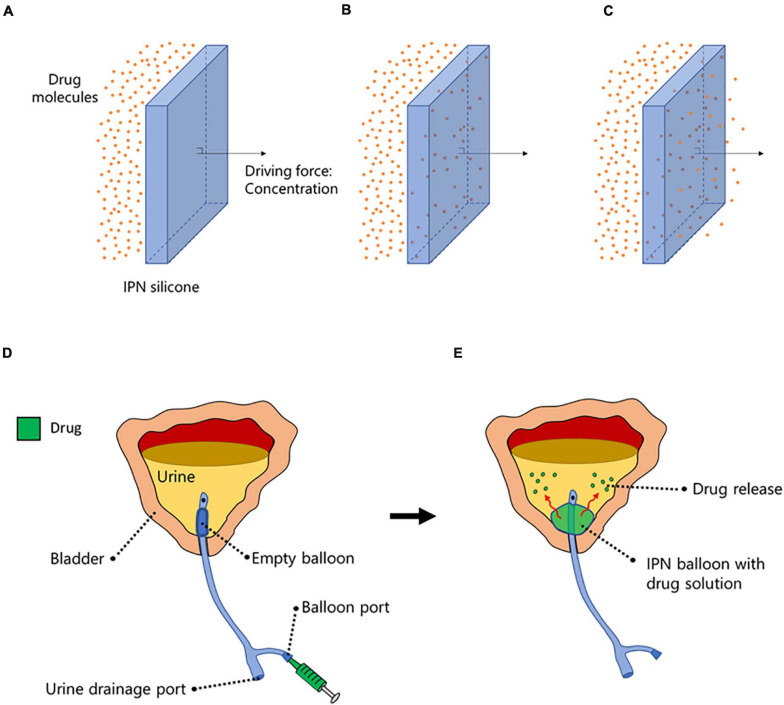
A conceptual schematic. **(A–C)** The interpenetrating polymer network (IPN)-silicone facilitates concentration-dependent drug molecule migration from one side of the material to the other. **(D)** When implemented into a Foley-type catheter balloon, the balloon is inflated with a drug solution through the balloon port. **(E)** Once inflated, the balloon provides dual functionality by (i) ensuring anchorage of the catheter within the bladder and (ii) providing a drug reservoir capacity for controlled drug-release into the urine.

Conventional Foley-type catheters are constructed with a balloon at the tip of the catheter, which, after insertion into the bladder, is inflated with liquid to ensure anchorage within the bladder. For the first time, we have succeeded in adding a second function to the balloon by converting the balloon material to an IPN that allows water-soluble drugs to diffuse across the balloon wall. The IPN-modified balloon can be inflated with an antibiotic solution to enable local, controlled release of antibiotics directly to the bladder urine, thus preventing bacterial colonization and CAUTI. A conceptual schematic is illustrated in Figure 1.

The concept was tested in a large animal model of CAUTI suited for testing full-sized urinary catheters. The IPN catheter (hereafter IPN-C) as well as a traditional antimicrobial-coated catheter in the form of the commercially available and widely used Bactiguard Infection Prevention Foley catheter (hereafter BIP-C) were tested against an experimental inoculation with UPEC. The study showed therapeutic drug release from the IPN balloon that successfully prevented experimental bladder infection in pigs.

## Materials and Methods

### Materials

All chemicals are supplied by Sigma-Aldrich (Søborg, Denmark, Germany) unless otherwise stated. The IPN catheter balloon host polymer was made from polymer Square SR330MA/MB silicone supplied by Shenzhen Square Silicone Materials Co., Ltd. (Shenzhen, China). The IPN guest polymer was made from 97% 2-hydroxyethyl methacrylate (HEMA) with 200 ppm monomethyl ether hydroquinone (MEHQ) as inhibitor and ethylene glycol methyl ether acrylate with (EGMEA, average MW = 480 g/mol) with 100 ppm butylated hydroxytoluene (BHT) and 100 ppm MEHQ as inhibitor. The inhibitors were removed by passing both monomers through an inhibitor remover column packed with quatamine divinylbenzene/styrene copolymer beads on Cl^–^ ion form (De-Hibit 200) supplied by Polysciences (Warrington, PA, United States). HEMA was further purified by distillation at reduced pressure, and the fraction at 67°C and 3.5 mbar was collected. Both monomers were stored at 5°C.

Ethylene glycol dimethacrylate (EGDMA) was used as crosslinker for the guest polymer in the IPN. Ethanol (EtOH) (99.8%) supplied by VWR (Søborg, Denmark) and tetrahydrofuran (THF) were used as cosolvents as received. Anaerobe CO_2_ 4.0 was supplied by Aga Denmark A/S (Ballerup, Denmark) and used as received.

Diethyl peroxydicarbonate (DEPDC) was used as initiator and synthesized by reacting 12.00 ml ethyl chloroformate (122.5 mmol) with 6.64 ml 30% H_2_O_2_ (58.59 mmol) and 24.00 ml 5M NaOH (120 mmol) in 100 ml precooled demineralized water under stirring (Xu et al., 2007). The reactants were added drop by drop to ensure that the temperature never exceeded 10°C. After gentle stirring for another 10 min, 50 ml of precooled hexane was added to extract DEPDC under increased stirring speed for 5 min. The mixture was transferred to a separation funnel, and the organic phase was collected. The separation was done twice. The produced DEPDC was stored in hexane at −18°C. The concentration of DEPDC in hexane was measured by titration with iodine to 0.2M according to American Society for Testing and Materials (ASTM) method E298-17a. The initiator mixture was regularly examined by the semiquantitative Quantofix method using peroxide test stick supplied by Macherey-Nagel (Düren, Germany).

### Fabrication of the Silicone-IPN Balloon

The host silicone polymer was hot plate molded to hollow cylinders (balloons) with length of 4 cm, inner diameter of 4.0 mm, and wall thickness of 0.5 mm. One hundred specimens (approximate weight of 14 g) were organized in a stainless-steel grid and placed in a 1-L reactor (1L, BC-2, HiP, Erie, PA, United States). HEMA (100.0 ml), 100.0 ml PEGMEA, 6.0 ml EGDMA, 50.0 ml 0.2 M DEPDC in hexane, 70.0 ml EtOH, and 70.0 ml THF were mixed for 20 min in a separate beaker before adding to the reactor. The solution was continuously stirred in the reactor using a magnet stirrer (Ret Basic, IKA, Staufen, Germany). The reactor was heated by a custom-made heating jacket and pressurized by a P-50 electrical-driven high-pressure pump (Thar Designs Inc., Pittsburgh, PA, United States). CO_2_ was added to the reactor to a pressure of 300 bar at 40°C. During 19-h polymerization time, monomers diffuse into the swollen silicone, polymerize, and crosslink. Following depressurization, the IPN samples were collected and excess polymer removed by rinsing in tap water. Residual monomer and non-crosslinked polymer were extracted by placing the IPNs in 96% EtOH for 7 days. The IPN specimens were then allowed to dry before the hydrogel content was estimated by gravimetric analysis.

### Catheter Assembly and Sterilization

The IPN balloon was glued onto standard 14-Fr Foley silicone catheters, packaged, and sterilized in ethylene oxide.

### Cather Inflation Capacity

Five IPN catheters and five standard silicone catheters (Rüsch) were submerged in water and inflated with water. The maximum volume before burst was recorded. The test was performed with dry IPN-C’s as well as IPN-C’s presoaked for 3 h in water or EtOH.

### Confocal Laser Scanning Microscopy of Fluorescein Isothiocyanate Migration

An IPN-C was inflated with 5 ml 2.4 mM fluorescein (Fluka, St. Louis, MO, United States) in water and placed freely on a cover glass slip. The balloon material was visualized with a Zeiss LSM 510 confocal laser scanning microscope using a C-Apochromat 63x/1.2W objective and LP 505 filter. Z-stacks of 10 frames (146.2 × 146.2 × 2.0 μm) for a total stack height of 18 μm were obtained at 0, 25, 43, and 72 min.

### Release Assay for Drug Screening

*In vitro* release of drug candidates was performed by inflating IPN-C balloons with 10 ml of drug in demineralized water (25 mg ml^–1^) and submerging it in 200 ml demineralized water at 37°C, which was replaced every 24 h. Drug concentration in the release media was analyzed on an Evolution 220 UV-Visible spectrophotometer from Thermo Fisher Scientific (Waltham, MA, United States) using the INSIGHT software four times daily for 4 days. Average release rate was estimated by linear regression of the data from the four measurements per day.

### Bacterial Strains and Growth Media

For the animal infection experiments, the standard cystitis strain UTI89 was used (Hung et al., 2009; Nielsen et al., 2019). UTI89 was preincubated as described by Hung et al. (2009) to ensure optimal type-1 pili expression. In short, 25 ml of Lysogeny Broth (LB) was inoculated with an agar plate colony and incubated ON. The next day, 25 μl of bacterial suspension was transferred to a new broth of 25 ml LB. On the day of infection, the bacterial inoculum was prepared immediately before inoculation by centrifuging the suspension at 2,500 × *g* for 20 min and resuspending the pellet in sterile NaCl. This suspension was adjusted to an optical density of 1.0 at 600 nm, which approximately equals 1 × 10^9^ colony forming units (CFU) ml^–1^. The suspension was serially diluted to reach a final concentration of 1 × 10^5^ CFU ml^–1^.

For the *in vitro* studies of long-term release (below) and minimum inhibitory concentration (below), a plate colony of UPEC strain UTI89 was suspended in sterile NaCl and adjusted to McFarland = 0.5. From this suspension, 100 μl was transferred to 9.9 ml of Mueller Hinton II Bouillon (SSI Diagnostica, Hillerød, Denmark) or serum bouillon (SSI Diagnostica) for minimum inhibitory concentration (MIC) determination.

### Minimum Inhibitory Concentration

All drugs were purchased from Merck and prepared in stock solutions: nitrofurantoin and nitrofurazone were dissolved in dimethyl sulfoxide (DMSO) (25 mg ml^–1^), sparfloxacin in DMSO and 0.1M NaOH (9.6 mg ml^–1^), and chlorhexidine in water (20% solution). All drugs were further diluted in water for final concentrations of 64 μg ml^–1^. From here, 200 μl was transferred to a well in a sterile 96 round-bottom well plate. The following 11 wells in each row were added 100 μl of serum bouillon. For twofold serial dilution, 100 μl was transferred from the first well to the second and so on. One hundred microliters was discarded from the last well. Lastly, 100 μl of bacterial suspension in serum bouillon was added to each well. The lowest concentration of antibiotic agent resulting in no visual growth after incubation was considered the MIC.

### Long-Term Functional Release Assay

The balloons of IPN-Cs and standard 14 Fr silicone Foley catheters (Rüsch) were loaded with 8 ml of sparfloxacin suspension (50 mg ml^–1^) and each submerged into 1 L of sterile saline (0.9% NaCl). Every other day, NaCl samples were collected and the media was changed. To measure the antibacterial activity, 500 μl of NaCl samples was inoculated with 500 μl of UTI89 suspension in Mueller Hinton II bouillon and incubated ON. Viable bacterial counts were determined by serial dilution on agar plates. A 99.9% reduction in CFU was considered the minimum bactericidal concentration.

### Animals

A total of eight female domestic pigs (Landrace/Yorkshire) with an average initial weight of 25 kg were obtained from a pig herd (Kokkenborg ApS, Stenstrup, Denmark) with the highest health status according to the Danish SPF system (Red SPF-X, Salmonella index 0.0). The animals were kept in standard 3-m^2^ pens provided with sawdust bedding and straw and fed with standard feed and *ad libitum* water. Social and material (ball) enrichment was provided to maintain welfare. Upon arrival, the pigs were examined for signs of infection or other illness by a veterinarian and animal technicians. The pigs were allowed to acclimatize for 7 days before they were trained for 14 days to handle human touch around the site of the catheter. Animals were attended at least twice daily. During the experiment, the pigs were housed individually to avoid untimely catheter discontinuation. A protocol for humane endpoints was set up, but no animals were untimely sacrificed due to unexpected discomforts or illness. All animal experiments were carried out according to the EU directive 2010/63/EU for animal experiments and were approved by the Danish Animal Experiments Inspectorate, license number 2019-15-0201-01626.

### Porcine CAUTI Model

**FIGURE 2:**
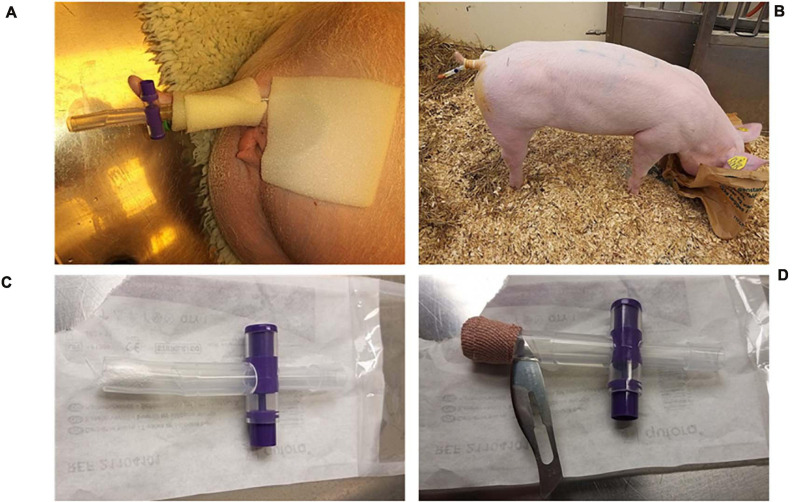
Catheter fixation. **(A)** External fixation of the catheter. **(B)** The catheters were easily accessible, and due to proper training prior to the experiment, urine samples could be collected without the need for sedation. **(C)** To limit contamination of the catheters from the environment, the catheter end was always covered with a replaceable catheter valve that was either closed when accumulating urine for sampling **(A)** or open and sealed with gauze swaps **(C)** and secured with tape **(D)** so that urine could slowly drip through. **(D)** A secondary non-return valve was made by cutting a thin slit in the silicone right above the gauze. The catheter valve was always discarded before urine sampling and replaced with a sterile valve after sampling.

The porcine CAUTI model was developed based on Nielsen et al. (2019). The intervention group consisted of three pigs that were catheterized with a 14 Fr IPN-C, and the balloon was inflated with 8 ml 50 mg ml^–1^ sparfloxacin (Sigma) suspension. In the benchmark test, three pigs were catheterized with a BIP-C with 8 ml of saline in the balloon. In the control group, two pigs were catheterized with an IPN-C, but the balloon was filled with 8 ml of sterile water. A baseline urine sample was collected before the catheter balloon was filled to confirm sterility of the urine. The catheters were connected to a replaceable catheter valve and fixed to the tail using tape (Figure 2).

In order to collect sufficient volumes of urine, the catheter valves were closed for 2 h prior to urine sampling. During urine sampling, the valve was removed, and the initial 10 ml of urine was discarded, after which the urine was sampled directly from the catheter with a syringe. A new sterile valve was inserted afterward. In between sampling, the valves remained opened, but the tip of the valve was closed with gauze and secured with tape so that the catheter was guarded against contamination while the urine could slowly diffuse through the swap and tape (Figures 2C,D). For safety reasons, a secondary non-return valve was constructed by cutting a slit (length = 1 cm) in the replaceable catheter valve with a sterile scalpel (Figure 2D). The nature of the silicone keeps the slit closed so that dirt and other contaminants cannot enter, but urine may pass through if the pressure rises moderately as a result of accumulating urine.

The day after catheterization, the catheter valves were closed for 2 h prior to inoculation with 10 ml bacterial suspension (10^5^ CFU ml^–1^) followed by a 1-h void restriction (closed valve) for effective infection of the bladder. After the void restriction period, the bladder was emptied.

For sedation, pigs were premedicated with medetomidine (Cepetor, 0.12 mg kg^–1^), butorphanol (Butomidor, 0.2 mg kg^–1^), and Midazolam (Midazolam, 0.1 mg kg^–1^). Anesthesia was induced and maintained on 10 mg ml^–1^ propofol. As soon as propofol was initiated, the pigs were immediately given IM antisedan (0.2 mg kg^–1^) to reverse the α_2_-adrenergic diuretic effects of medetomidine. Blood samples were collected from the jugular vein during sedation.

At the last day of the experiment, pigs were sedated, and a urine sample was collected, after which the catheters were removed and transferred to a sterile sheet. Specimens were cut out in 10-mm pieces from predefined sites along the length of the catheter. Care was taken not to puncture the balloon or balloon channel to prevent release of residual sparfloxacin. Hereafter, the pigs were sacrificed with pentobarbital (140 mg kg^–1^), and urinary bladders were aseptically removed within 10 min *post mortem*.

### Urine and Blood Sample Analysis

Urine samples were analyzed for urine specific gravity (USG) and serially diluted and plated on LB agar (SSI Diagnostica) to quantify the level of bacteriuria. Bacterial species were identified with matrix-assisted laser desorption ionization time of flight (MALDI-TOF) (Bruker, Billerica, MA, United States), and environmental contaminants, i.e., bacterial species other than *E. coli*, were excluded from further analysis. Urine sparfloxacin concentration was assessed indirectly by determining the concentration factor relative to MIC for UTI89. To do so, urine samples were diluted in twofold serial dilution and inoculated with UTI89 similar to MIC determination (described above). The number of dilutions before growth was considered the concentration factor.

Serum biochemical parameters [white blood cell (WBC), differential cell count, red blood cell (RBC), hematocrit, hemoglobin, platelet, mean platelet volume (MPV)] were analyzed using a SCIL Vet animal blood counter (ABX Diagnostics, Montpellier, France). Blood samples were centrifuged at 2,500 × *g* for 10 min, and plasma was retrieved and stored at −80°C. Plasma was analyzed for C-reactive protein (CRP) with CRP Pig ELISA Kit (Abcam ab205089).

### Catheter Analysis

Catheter pieces were sonicated for 5 min in 3 ml sterile NaCl before plating in serial dilutions on LB agar to quantify CFU. Further catheter pieces including the balloon were stained using the LIVE/DEAD BacLight Bacterial Viability Kit (Invitrogen, Carlsbad, CA, United States), fixed in 10% formalin and visualized on an Olympus FV1000MPE confocal laser scanning microscope using an XLUMPlanFI × 20/0.95 W objective and Olympus FV10−ASW software.

### Gentamicin Protection Assays

After euthanization of the pigs, whole bladders were aseptically removed and immediately transferred to a sterile laminar airflow cabinet. Bladders were cut open at one side between the ureters, splayed, and gently washed in sterile saline to remove non-adherent bacteria. The bladders were then divided into smaller pieces by punching out disk-shaped specimens (Ø = 10 mm). Three randomly chosen samples were immediately homogenized using an Ultra-Turrax T25 rod disperser at 22,000 rpm (IKA), and the homogenate was serially diluted and plated on LB agar to quantify bacteria associated within the bladder tissue. To quantify intracellular bacteria alone, three other samples were incubated for 90 min in 37°C preheated Dulbecco’s modified Eagle’s medium (DMEM) supplemented with 10% fetal bovine serum and containing 300 μg ml^–1^ gentamicin, which only kills extracellular bacteria. The gentamicin-treated samples were then thoroughly rinsed in saline, homogenized, and plated as above. To verify that no leftovers of gentamicin or sparfloxacin were present, aliquots of the homogenates were sterile filtered and inoculated with UTI89 for verification of growth.

### Statistical Analysis

All statistical analyses were performed with GraphPad Prism software version 8.0.2. Analysis of more than two groups were performed with one-way ANOVA using Brown–Forsythe’s test for variance followed by Tukey’s multiple comparisons test.

## Results

### Assessment of Balloon Basic Functionality

According to the ISO20696 Annex D, a Charrière 14 catheter balloon must be inflatable to × 1.2 its intended volume (i.e., 12 ml) to ensure that the balloon does not burst inside the bladder. To assess the strength and flexibility of the IPN material to function as an expandable balloon, five catheters were inflated with saline, and the maximum volume before burst was recorded. The IPN material could be inflated on average with 30 ml (SD, 0.0) before burst compared to 35 ml (SD, 5.2) for standard silicone catheter (Rüsch) (Supplementary Figure 1).

**FIGURE 3:**
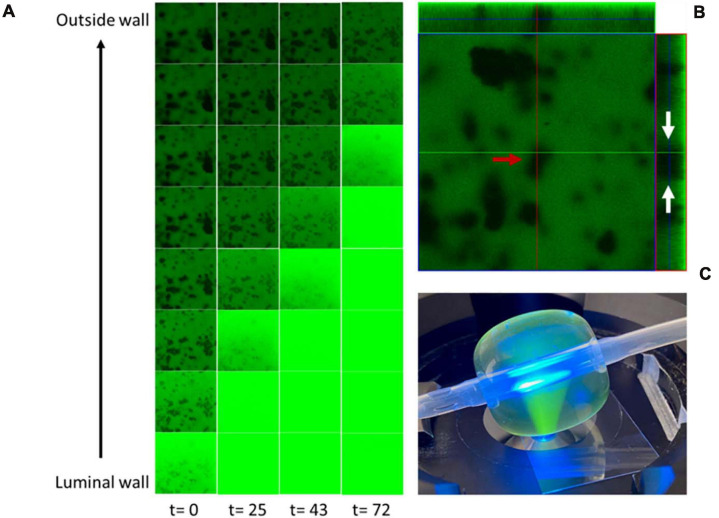
Fluorescein isothiocyanate (FITC) migration through the interpenetrating polymer network (IPN) balloon. **(A)** Time lapse microscopy recorded at 0, 25, 43, and 72 min shows a time-dependent drug migration. **(B)** Z-stack at t = 0 shows a heterogenous material with large areas of hydrogel networks capable of absorbing FITC (green areas) and smaller silicone domains that remain unloaded (red arrows) and extends through the material (white arrows). **(C)** Experimental setup showing the time-lapse confocal laser scanning microscopy (CLSM) performed on an IPN catheter inflated with 5 ml of FITC. Frames in A- and Z-stack **(B)** are 146 μm × 146 μm. t, time in minutes.

Solute migration through the balloon IPN was visualized by time-lapse confocal laser scanning microscopy (CLSM) of an IPN-C balloon loaded with a saturated fluorescein isothiocyanate (FITC) solution (Figure 3C). FITC was observed to migrate steadily from the luminal wall toward the outer wall, thus saturating the material indicated by increased fluorescence (Figure 3A). Parallel analysis of standard control catheters loaded with the same solution yielded no detectable signal from the balloon material over the same period of time, indicating no diffusion of drug (data not shown). The CLSM analysis of the IPN balloon material revealed large areas of hydrogel networks with drug-loading properties [indicated by rapid FITC saturation (Figure 3B, green areas)] interlaced by smaller silicone domains (Figure 3B, red arrow) that absorbed fluorescein more slowly or not at all. Orthogonal imaging shows that hydrogel areas span across the balloon material (Figure 3B, white arrows).

### Drug Candidate Screening for IPN-C Release

A panel of conventional UTI antibiotics as well as selected disinfectants were tested *in vitro* as potential drug candidates for intravesical IPN-C delivery. This panel consisted of sparfloxacin, a broad spectrum fluoquinolone with antimicrobial effect against most uropathogens, as well as two nitrofurans, nitrofurantoin and nitrofurazone, the former being the most common choice of per oral CAUTI prophylactic. Nitrofurazone was selected, as this drug has previously been used as agent for slow release from catheters, while chlorhexidine represents a broadly used disinfectant not suitable for systemic treatment but which may be used locally (Desai et al., 2010). IPN-C balloons were filled with 10 ml of drug (25 mg ml^–1^) and submerged in 200 ml water. The drug concentration was measured daily concurrently with change in media and is summarized in Table 1. Nitrofurantoin and nitrofurazone was released in average rates of 19.68 and 47.84 μg/h, respectively, from day 1. In contrast, the release of sparfloxacin and chlorhexidine was characterized by a lag phase between 4 and 24 h for sparfloxacin and between 4 and 48 h for chlorhexidine. After the lag phase, sparfloxacin release averaged at 20.91 μg/h and chlorhexidine at 116.4 μg/h.

**TABLE 1:** Drug release rate.

Drug	Release rate 4 h (μg/h), [R^2^]	Release rate 24 h (μg/h), [R^2^]	Release rate 48 h (μg/h), [R^2^]	Release rate 72 h (μg/h), [R^2^]	Average (μg/h)
Nitrofurantoin	23.89 (0.9436)	14.08 (0.9975)	23.09 (0.9835)	17.64 (0.9971)	19.68
Nitrofurazone	54.51 (0.9727)	41.50 (0.9511)	54.71 (0.9872)	40.63 (0.9630)	47.84
Sparfloxacin	1.47 (0.1838)	19.03 (0.9893)	26.08 (0.9693)	17.62 (0.9740)	20.91^a^
Chlorhexidine	2.55 (0.1434)	29.96 (0.9922)	105.53 (0.9788)	126.74 (0.9757)	116.14^b^

*Experiments were performed in quadruplicates.*

*^*a*^Average calculated based on 24 and 72 h (steady-state release).*

*^*b*^Average calculated based on 48 and 72 h (steady-state release).*

Minimum inhibitory concentration was determined for every drug, and the results are summarized in Table 2. Although having a low water solubility, sparfloxacin was selected for subsequent *in vitro* and *in vivo* proof-of-concept experiments due to its low MIC value (Table 2) combined with a decent release rate over the IPN-C balloon.

**TABLE 2:** Drug characteristics and minimum inhibitory concentrations.

Drug	Chemical formula	Mw (g mol^–1^)	MIC (μg ml^–1^)	Log P	Lag phase, t_pen_ (h)	Water solubility (mg ml^–1^)
Nitrofurantoin	C_8_H_6_N_4_O_5_	238.16	4	−0.47	<1	0.08
Nitrofurazone	C_6_H_6_N_4_O_4_	198.14	8	0.23	<1	0.21
Sparfloxacin	C_1__9_H_2__2_F_2_N_4_O_3_	392.4	<0.016	2.5	4 < t_pen_ < 24	0.11
Chlorhexidine	C_2__2_H_3__0_Cl_2_N_10_	505.4	>64	0.08	4 < t_pen_ < 24	0.80

*Minimum inhibitory concentration (MIC) is measured for uropathogenic Escherichia coli, UTI89. t_*pen*_ = time to penetration (lag phase).*

### Sparfloxacin Is Released in Therapeutic Concentrations From the IPN-Balloon for Up to 30 Days

**FIGURE 4:**
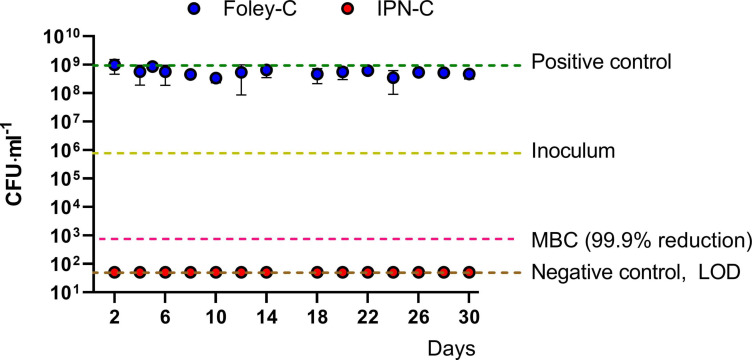
Functional release assay. Standard Foley catheters and interpenetrating polymer network (IPN) catheters were loaded with 8 ml of sparfloxacin 50 μg ml^–1^ and submerged into 1 L of sterile NaCl solution. Samples were collected from the media every other day after which the media was changed. Aliquots were inoculated with UTI89 and incubated ON to evaluate antibacterial activity. The suspensions were plated to quantify viable bacteria. Media retrieved from bottles with Foley catheters (blue dots) did not prevent bacterial growth as CFU reached levels similar to the positive control (saline + inoculum, green line). In contrast, no viable bacteria were detected in media from bottles with IPN catheters (red dots) for the monitoring period of 30 days. Data represents means ± SD from three independent experiments. MBC, minimum bactericidal concentration; LOD, limit of detection (50 CFU ml^–1^).

To assess the long-term prophylactic effect of the IPN-C concept, IPN-Cs and standard Foley catheters were loaded with 8 ml of sparfloxacin suspension (50 mg ml^–1^) and submerged in 1-L bottles with sterile NaCl solution. The bottles were then incubated at 37°C with media renewal each second day. Samples were collected regularly and inoculated with UTI89 suspension to assess antimicrobial effect. After 16 h incubation, aliquots were plated to quantify viable bacteria. The results are summarized in Figure 4. As expected, the standard Foley catheters did not release any sparfloxacin as indicated by the non-inhibitory activity toward UTI89 (Figure 4, blue dots). In contrast, no viable bacteria were detectable in the media from IPN-Cs (Figure 4, red dots), demonstrating that the sparfloxacin released from the IPN-C reached concentrations above the minimum bactericidal concentration. The total inhibition persisted throughout the experiment (30 days).

**FIGURE 5:**
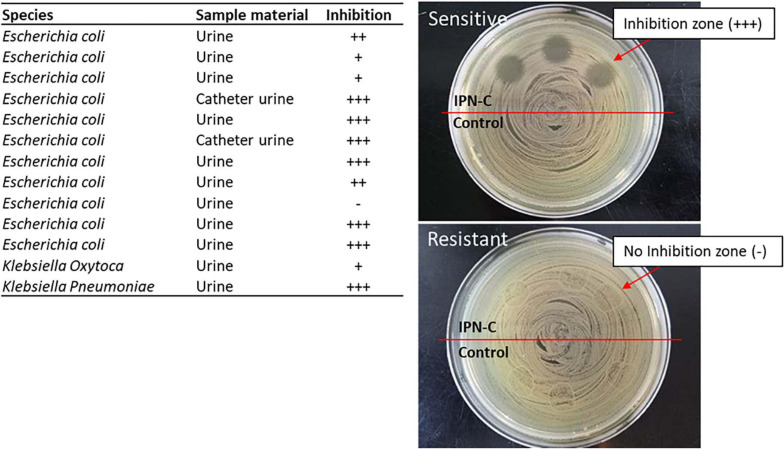
Inhibition test of released sparfloxacin against clinical uropathogenic bacterial isolates. Aliquots (20 μl) of NaCl from release assays (day 6) were placed on LB agar plates that had been homogeneously inoculated with clinically isolated uropathogens using a plate spreader. After ON incubation at 37°C, inhibition was graded as: no inhibition (−), inhibition zone with several single-colonies (+), inhibition zone with few single-colonies (++), inhibition zone with no single-colonies within the zone (+++). Sample material indicates whether the bacteria is isolated from urine samples or a catheter urine sample.

The reference UPEC strain UTI89 was highly sensitive toward sparfloxacin (MIC = < 0.016 μg ml^–1^, Table 2). To assess whether the sparfloxacin/IPN-C combination would be useful against other clinical uropathogenic strains as well, we investigated the inhibitory effect of the release media after 6 days against a panel of 13 *Escherichia coli* and *Klebsiella* spp. isolated from the urine of human patients. The media effectively inhibited 91% (10 of 11) of the *E. coli* isolates and 100% (2/2) of *Klebsiella* isolates (Figure 5).

### The IPN-C Effectively Prevents UPEC From Colonizing the Porcine Bladder During Experimental Inoculation

**FIGURE 6:**
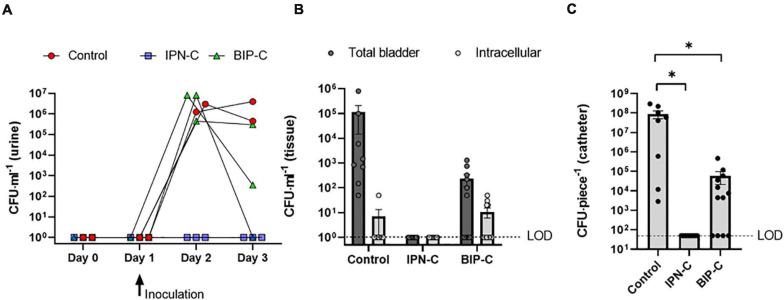
Bacterial burden of inoculated pigs. All controls (red circle) and all pigs with a BIP-C (green triangle) developed significant *E. coli* bacteriuria (>10^5^ CFU ml^–1^) 24 h after inoculation (day 2) **(A)**. On day 3, one pig in the BIP-C group had cleared the infection (sterile urine) while another had only minor bacteriuria (3.5 × 10^2^ CFU ml^–1^). *E. coli* remained undetectable throughout the experiment in urine samples from pigs with an interpenetrating polymer network interpenetrating polymer network (IPN)-C (blue square). Bladder-wall-associated bacteria were present in control pigs and two of three pigs in the BIP-C group but were undetectable in the IPN-C group **(B)**. Intracellular bacteria were few and, in most cases, undetectable for all groups. No *E. coli* were detectable on the IPN-C catheters **(C)**. BIP-C catheters revealed significantly less *E. coli* biofilm compared to control, and one catheter was completely devoid of *E. coli* colonization **(C)**. Bars represents means ± standard error of the mean. **(B,C)** Represents data from four tissue/catheter samples from each animal. CFU numbers in C represents adherent bacteria from 10 mm catheter pieces. *P = 0.0032, one-way ANOVA with Tukey’s multiple comparisons test. CFU, colony forming units; LOD, limit of detection was **(A,B)** 10 CFU ml^–1^ and **(C)** 50 CFU ml^–1^.

The IPN-C was used in a challenge of prophylactic intervention in a porcine model of CAUTI against a control and a BIP-C (benchmark) group. Eight pigs were catheterized on day 0 with either IPN-C with sparfloxacin (*n* = 3), IPN-C with saline (control, *n* = 2), or BIP-C with saline (*n* = 3). On day 1, all pigs were inoculated via the catheter with 10 ml UPEC suspension (10^5^ CFU ml^–1^), and urine CFU was monitored in daily urine samples until termination of the experiment on day 3. The results are summarized in Figure 6A. The control group and the BIP-C group developed significant *E. coli* bacteriuria (>10^5^ CFU ml^–1^) within 24 h after inoculation (day 2). The control group remained heavily colonized on day 3, however, while one pig in the BIP-C group remained heavily colonized (>10^5^ CFU ml^–1^), another pig from the BIP-C group had cleared the infection (sterile urine), and the third pig had reduced the level of bacteriuria to below clinical significant levels (<10^3^ CFU ml^–1^). In the IPN-C group, no UPECs were detectable in the urine samples during the experiment, indicating that sparfloxacin had effectively diffused over the IPN balloon wall reaching therapeutic concentrations in the porcine bladder. Environmental contaminants were occasionally observed to some extent in urine samples and were most evident in IPN-C pigs where UPEC growth was inhibited. In the control and BIP-C groups, contaminants were negligible, as they only comprised a fraction of the total CFU. Bacterial contaminants were identified with MALDI-TOF, and a list of identified species are summarized in Supplementary Figure 2. *Enterococcus faecalis* was the only potential urinary pathogen detected among these species. *In vivo* drug release was further assessed by analysis of sparfloxacin in the collected urine samples, showing concentrations of 32–64 × MIC (data not shown). The urine production, which directly influences drug concentrations of the bladder assuming zero-order release kinetics independent of the urine concentration, was indirectly assessed by continuously determining the USG of urine samples, which were found to be on average 1.015 (range, 1.0049–1.026) and hence well within the range of urine in humans (Parfentjev and Perlzweig, 1933).

### The IPN-C Catheter Protects Against Sessile Colonization of the Bladder Tissue and Catheter Surface

**FIGURE 7:**
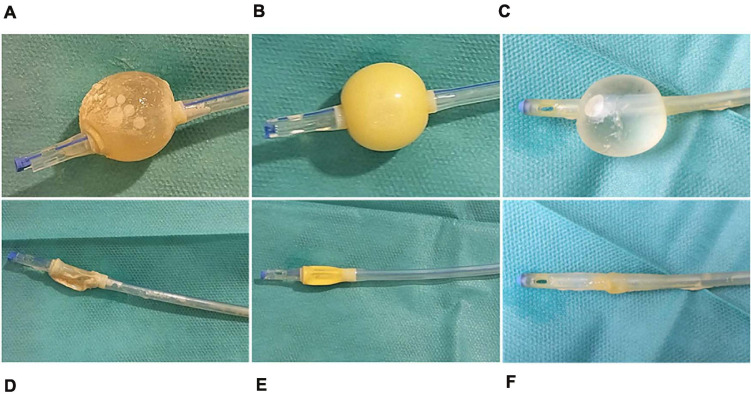
Catheters posttreatment. Catheters were removed at termination for visual inspection of the balloon and shaft. **(A,D)** Control; **(B,E)** interpenetrating polymer network (IPN)-C; **(C,F)** BIP-C.

**FIGURE 8:**
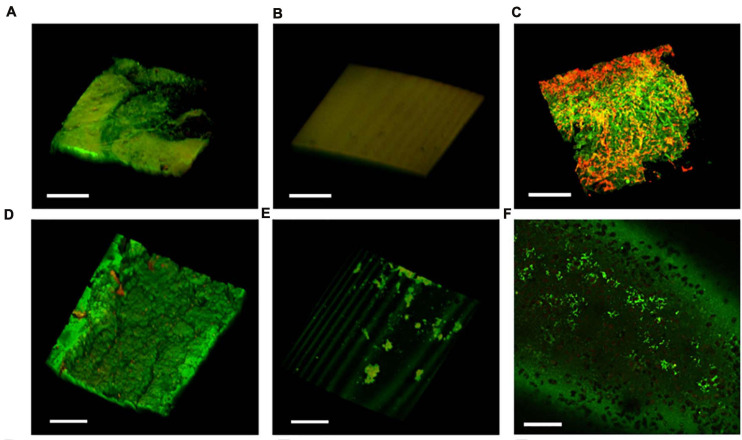
Bacterial adhesion and biofilm buildup on the catheter. Confocal laser scanning microscopy analysis of the catheter balloon (top row) and luminal surface of catheter shafts (bottom row) after termination on day 3. The control catheter showed widespread biofilm formation on the **(A)** balloon and **(D)** catheter shaft. **(B)** No bacteria were visible on the interpenetrating polymer network (IPN)-C balloons, **(E)** but colonies of environmental contaminants were occasionally observed on the IPN-C shafts. The BIP-Cs were characterized by moderate amounts of bacteria on the **(C)** balloon and **(F)** shafts. **(C)** BIP-Cs were also covered with considerable amounts of mucus and epithelial debris (red). Bacteria were stained with Filmtracer LIVE/DEAD stain. Scale bar = 100 μm.

On day 3 of the experiment, pigs were sedated, and the catheters were removed. Catheters from control animals were encrusted with biofilm and pus (Figures 7A,D), whereas IPN-C and BIP-C catheters appeared clean (Figures 7B,C,E,F). Quantification of adherent UPEC was performed by sonicating 10 mm cutout pieces from the catheter shaft in 3 ml saline solution and subsequently plating aliquots on agar. In the BIP-C group, mean catheter-adherent UPEC of 8.9 × 10^4^ CFU⋅piece^–1^ was significantly lower (*p* = 0.003) compared to controls (mean, 9.0 × 10^7^ CFU⋅piece^–1^) (Figure 6C). No UPEC were detected on IPN-C catheters (Figure 6C). This was further supported by CLSM analysis of the balloon and shafts (Figure 8). After removal of the catheters, the pigs were sacrificed, and whole bladders were aseptically removed within 10 min *post mortem*. Harvested bladders were split in smaller samples for quantification of tissue-adherent and intracellular bacterial populations using gentamicin-protection assays. The results are summarized in Figure 6B. Tissue-adherent bacteria were present in all control and BIP-C pigs, but intracellular bacteria were sparse and, in most cases, undetectable (>84% of samples) (Figure 6B). No tissue-adherent or intracellular UPEC population was detectable in the IPN-C group.

## Discussion

IPN as a material to facilitate controlled drug release has been studied extensively in pharmaceutical sciences over the past decades, and with general advances in polymer sciences, it has been a fundamental concept in a variety of novel drug delivery systems (Kurkuri and Aminabhavi, 2004; Agnihotri and Aminabhavi, 2005, 2006; Dragan, 2014; Lohani et al., 2014; Aminabhavi et al., 2015). Hydrogel IPNs have mainly been used to control drug release in tablet- and capsule-based formulations or in biodegradable implants with local drug-delivery properties (Changez et al., 2004, 2005; Lohani et al., 2014; Aminabhavi et al., 2015). Due to limitations in the manufacturing process, the IPN concepts explored in the latter are often microscale particle based. As we recently showed, IPN can, however, also be manufactured as a non-degradable base material in flexible medical devices, providing a reservoir in the bulk of the material for controlled release of drugs (Stenger et al., 2016; Klein et al., 2017).

Here, we used the same sequential IPN technology on thin, low shore hardness silicone and succeeded in fabricating an IPN material that possesses the strength and flexibility to function as an inflatable catheter balloon while retaining the controlled drug-release properties of the hydrogel IPN structure. The material was used in the balloon of functional Foley catheters, a catheter type that has been the most widely used since its invention 1929. In the Foley design, the balloon keeps the catheter locked in place upon inflation with glycerol solutions *in situ*. By converting the balloon into a drug-permeable membrane, new functionality is achieved enabling slow-release drug administration locally in the urinary tract.

Using the catheter balloon lumen for drug storage essentially provides an unlimited reservoir capacity. While drug coatings on antimicrobial catheters degenerate over time and often fail to significantly reduce the risk of CAUTI, drugs administered over the balloon wall retains the same daily release rate and can be replenished if needed, without the need of replacing the catheter (Pickard et al., 2012a,b; Björling et al., 2018).

Despite an initial lag phase of 24 h before reaching steady-state release, we showed that after 30 days, the antibiotic sparfloxacin was still released in therapeutic concentrations *in vitro*, thus demonstrating the potential of the balloon reservoir for long-term prophylaxis of CAUTI.

Although previous studies have tested slow-release hydrogel IPNs *in vitro*, few have taken the important step further and evaluated IPN concepts *in vivo* (Aminabhavi et al., 2015; Zizovic, 2020). Here, we established, to the best of knowledge the first large-animal model of CAUTI as an experimental platform to test novel catheter concepts. Existing models of chronic catheterization in pigs, which are used in livestock sciences and never before used to study CAUTI, involve the placement of animals largely fixed in metabolic cages, without bedding and social isolation, which results in negative behavioral and physiological reactions (Herskin and Jensen, 2000; Obernier and Baldwin, 2006; Nørgaard et al., 2010). Animals are found not to acclimatize to metabolic cages, and the entailing metabolic changes warrant caution when interpreting data, as their condition cannot be considered representative for normal physiology (Kalliokoski et al., 2013). With the currently presented large-animal CAUTI model, we show that it is possible to maintain the animals catheterized while allowing them to move freely in pens. Evaluation of the general wellbeing based on behavior and blood biomarkers of inflammation did not indicate signs of social or physiological stress. Overall, this suggest that results obtained with this model more accurately reflect the natural course of disease compared to models based on metabolic cages.

We used the model to test the capacity of IPN-C to prevent colonization of the device and the bladder when challenged with UPEC. Pigs with control catheters developed significant bacteriuria and biofilm on catheters, congruent with previous studies from our porcine cystitis model where pigs consequently become infected upon inoculation (Nielsen et al., 2019). The IPN-C succeeds in preventing CAUTI, as no viable UPECs were detected not only on the catheter surface but also in the urine samples and bladder wall. This verifies that the hydrogel IPN provides an adequate drug release to maintain therapeutic concentrations within the bladder lumen *in vivo.*

The BIP-C, which is validated in clinical trials and so far sold in over 200 million copies, was used as a benchmark test (Lederer et al., 2014). The technology behind the anti-infective effects of the BIP catheter is based on an alloy coating of gold, silver, and palladium that, upon contact with fluids, produces a galvanic reaction that repels bacteria and thus prevents their attachment to the catheter surface (Björling et al., 2018). Indeed, adherent bacteria were significantly reduced in BIP-C compared to that in control, but BIP-C failed to prevent the experimental infection in this model, as all pigs in the BIP-C group developed significant bacteriuria (>10^5^ CFU ml^–1^) 24 h postinfection (hpi). Surprisingly, however, some pigs in the BIP-C group were found to partly clear the infection over time, as bacteriuria was significantly reduced in the urine samples at 48 hpi, thus demonstrating that the antibacterial effects of the BIP-C are not exclusive to the catheter surface but actively suppress the UPEC population inside the bladder lumen as well. This may be a result of metal-ion leakage, particularly silver, which previously has been reported to be released in small amounts from a similar coating used in Bactiguard Infection Protection central venous catheters in human patients (Björling et al., 2018).

Being the first large-animal model of CAUTI, relevant results were obtained pertaining to UTI and CAUTI infection pathogenesis that deserve mentioning here. Extensive research in UPEC pathogenesis over the last two decades has documented an intracellular subpopulation of UPEC during UTI (Justice et al., 2004). In murine models, these intracellular colonies may survive antibiotic treatment and seed recurrent infections (Kerrn et al., 2005). Intracellular UPECs have not been convincingly demonstrated in human bladders, and their implication for recurrent UTI is debated (Kaye and Sobel, 2014; Köves and Wullt, 2016). The detection of viable UPEC in bladder tissues from control and BIP-C pigs after gentamicin treatment *ex vivo* indicates that UPEC may translocate to intracellular niches during infection of larger mammals. Consistent with previous studies in human bladder and vaginal cell cultures, we found, however, the intracellular population to be very limited (10–100 CFU/cm^2^ bladder-epithelial surface) (Stærk et al., 2016; Brannon et al., 2020). This contrasts with the general observations in murine UTI models, where intracellular bacterial communities harboring thousands of bacteria in a single bladder epithelial cell are frequently observed (Anderson et al., 2003; Justice et al., 2004, 2006). The cell-penetrating properties of sparfloxacin may explain why the bladder tissue of IPN-C pigs was completely cleared of intracellular UPEC. An alternative explanation is that the bacteria were immediately killed when introduced to the urine upon inoculation and never successfully colonized the bladder tissue.

Although our results show that administration of sparfloxacin via the IPN-C concept holds promise against CAUTI, one main weakness of the IPN-C concept is the spectrum of antibacterial effect limited to bacterial species sensitive to the drug in use. This was demonstrated by fluctuating environmental contaminants in the IPN-C group (Supplementary Figure 2). Sparfloxacin is a broad-spectrum fluoroquinolone with high antibacterial effect against the most common etiological agents of CAUTI, Enterobacteriaceae and *Pseudomonas* spp., and some effect against *E. faecalis* and *Staphylococcus aureus* (Piddock and Zhu, 1991; Rotstein et al., 1993). However, sparfloxacin is ineffective against *Candida* spp. and bacteria with acquired resistance that unfortunately occur quite frequently as a result of fluoroquinolones, mainly ciprofloxacin and ofloxacin, being widely used globally in treating UTIs and other infectious diseases (Hooper and Jacoby, 2015). No Enterobacteriaceae or *Pseudomonas* spp. were detected as contaminants; however, in one IPN-C pig, *E. faecalis* was found to some extent on day 2 and onward, suggesting that the accumulated sparfloxacin concentration in the porcine bladder was above the MIC for Enterobacteriaceae but below the MIC for *E. faecalis* that generally are less susceptible to sparfloxacin compared to Enterobacteriaceae such as *E. coli* (Cotter and Adley, 2001).

Topical prophylaxis is a growing trend in the management strategies of recurrent UTIs (rUTIs). Although Uro-Trainer polyhexanide, a novel routine decolonization solution for indwelling urinary catheters, is shown to reduce bacterial loads on catheters *in vitro*, the concept still lacks clinical validation (Brill et al., 2018). However, frequent intravesical instillations of gentamicin is an emerging intervention for rUTI prophylaxis in urological departments (Stalenhoef et al., 2019). The bactericidal activity of gentamicin is concentration dependent and increases with increasing peak concentrations, making gentamicin a well-suited antibiotic for intravesical instillations where a single large dose is administered with long intervals (Ambrose et al., 2007; Krause et al., 2016). Although gentamicin is an excellent UTI drug with a broad effect against most uropathogens, the effect of intravesical instillations are limited to sensitive strains, and gentamicin does not kill intracellular bacteria, which are suggested to contribute to rUTI (Rosen et al., 2007; Krause et al., 2016; Liu et al., 2016). The long intervals, typically 1–4 weeks, between the gentamicin instillations make it difficult to substitute gentamicin with other conventional UTI antibiotics such as nitrofurans, sulfonamides, β-lactams, or cephalosporins, as the bactericidal activity of these drugs are time dependent, thus benefitting from numerous administrations of low concentrations over time (Ambrose et al., 2007; Stalenhoef et al., 2019). The slow-release properties of the IPN-C is well-suited for the delivery of such time-dependent antibiotics, making the IPN-C a flexible intervention with the potential of substituting sparfloxacin with a single drug or combination of several suitable drugs that better target the microbial flora of an individual patient or a particular environment, such as an intensive care unit where antimicrobial resistance is high (Zhanel et al., 2008).

The dependence of antibiotic usage for IPN-C to function as an anti-infective intervention raises concerns about the development of antibiotic-resistant pathogens. The IPN-C concept may therefore be reserved for particularly susceptible patients as an alternative to protracted oral prophylaxis, which is associated with systemic adverse effects and the development of resistant strains of microbes, or as short-term postoperative prophylaxis following urological surgery (Morton et al., 2002; Fisher et al., 2018). In the animal experiments in this study, 500–1,000-fold less antibiotic was released to the bladder per day compared to the amount of drug ingested during standard per oral treatment with a similar fluoroquinolone (ciprofloxacin), indicating the potential for drastically reducing antibiotic consumption (assuming that drugs remaining in the balloon are retrieved and destroyed). Most antibiotics exert a serious disruptive effect on the gut microbiota that ultimately drives the emergence of antibiotic resistant pathogens (Carlet, 2012). Unlike per oral administration, the topical treatment with the IPN-C bypasses the gastrointestinal tract avoiding gut microbiota disruption, potentially reducing the contribution to antibiotic resistance. The beneficial impact of local bladder treatment on antibiotic resistance is supported in a recent study where intravesical instillations of gentamicin reduced the rate of multidrug-resistant pathogens by 70% in treated patients (Stalenhoef et al., 2019).

The sparfloxacin concentrations achieved in the porcine experiments roughly corresponded to the mean human urine peak concentration following a 400-mg per oral dose, which is the recommended therapeutic regimen (Montay, 1996). However, the absence of gastrointestinal drug absorption and systemic dissemination is likely to reduce adverse effects otherwise associated with per oral treatment, particularly nausea and diarrhea. Likewise, a wide spectrum of drugs that cannot be given orally, including certain disinfectants, may be administered locally via IPN-C in the bladder since toxic reactions in other tissues are avoided. The effective release of chlorhexidine over the IPN-C balloon as shown in this study shows that such an approach is feasible. Use of disinfectants and other non-antibiotics that are unsuited for per oral or intravenous treatment may indeed help combat multiple drug resistance due to their mode of action and local delivery.

In conclusion, we explored the use of IPNs in the Foley urinary catheter balloon material as a means of providing controlled drug release functionality for efficient intravesical UTI treatment. Drug release properties were demonstrated and analyzed *in vitro*, and proof of concept was obtained in a novel large-animal model of CAUTI. The study demonstrates the potential of the IPN-C as a novel concept for drug delivery in the bladder that may be used to reduce the risk of CAUTI in catheterized individuals. In perspective, the antimicrobial concept of IPN-C can potentially be adapted to other catheters including nephrostomy catheters that are also encumbered by high risk of infection (Ramstedt et al., 2019).

## Data Availability Statement

The raw data supporting the conclusions of this article will be made available by the authors, without undue reservation.

## Ethics Statement

The animal study was reviewed and approved by the Danish Animal Experiments Inspectorate.

## Author Contributions

KS: conceptualization, investigation, data curation, formal analysis, methodology, project administration, and writing—original draft preparation. RG: investigation, data curation, and writing—review and editing. YP: data curation, resources, and writing—review and editing. HK: conceptualization and writing—review and editing. LL: conceptualization, methodology, and writing—review and editing. MA: conceptualization, investigation, data curation, funding acquisition, and writing—review and editing. PT: conceptualization, funding acquisition, writing—review and editing. TA: conceptualization, data curation, investigation, methodology, project administration, funding acquisition, supervision, and writing—review and editing. All authors contributed to the article and approved the submitted version.

## Conflict of Interest

MA and PT are affiliated with Biomodics ApS that develops the IPN-C for commercialization. MA and PT have contributed with chemical data regarding release kinetics (Tables 1, 2), development of the IPN-C material (section Materials and Methods) and CLSM of FITC migration (Figure 3), but have not been involved in the analysis and interpretation of data from the functional release and *in vivo* experiments nor the final conclusions of this article. The remaining authors declare that the research was conducted in the absence of any commercial or financial relationships that could be construed as a potential conflict of interest.
