# Supplementary material for: A Novel Device-Integrated Drug Delivery System for Local Inhibition of Urinary Tract Infection
Source: Front Microbiol. 2021 Jun 25;12:685698. doi: 10.3389/fmicb.2021.685698 (PMC8267894; doi:10.3389/fmicb.2021.685698)

Supplementary Material

Supplementary figure S1

*Presoaked for 3 hours in water or EtOH respectively before test.

Supplementary figure S2. Environmental contaminants occasionally detected in urine samples from catheterized pigs


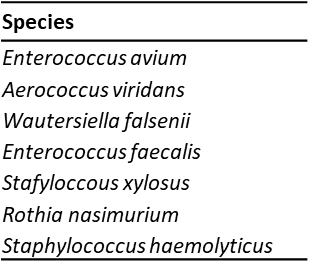

Supplement: Supplementary file 1 [file Table_1.DOCX]
